# Vegetation productivity responses to increased atmospheric vapor pressure deficit across the globe

**DOI:** 10.3389/fpls.2026.1886959

**Published:** 2026-08-12

**Authors:** Meiou Qin, Xiaotao Wang, Mengyuan Zhu, Peng Jiang, Rihong Wen, Binshuo Liu, Xingyang Li

**Affiliations:** 1 Liaoning Climate Center, Shenyang, China; 2 Liaoning Meteorological Service Center, Shenyang, China; 3 Liaoning Agricultural Meteorology and Satellite Remote Sensing Center, Shenyang, China; 4 Institute of Atmospheric Environment, China Meteorological Administration, Shenyang, China; 5 Liaoning Ecological Meteorology and Satellite Remote Sensing Center, Shenyang, China; 6 Tianjin Jinghai District Meteorological Bureau, Tianjin, China

**Keywords:** divergent responses, evapotranspiration, leaf area index, plant water-use strategy, vapor pressure deficit

## Abstract

**Introduction:**

Atmospheric vapor pressure deficit (VPD) has been identified as a key factor influencing vegetation productivity, with spatially divergent positive and negative effects observed across global ecosystems. However, the mechanisms underlying these heterogeneous responses remain insufficiently understood.

**Methods:**

Here, we integrated long-term global satellite observations with multiple statistical approaches to investigate the drivers of divergent vegetation responses to VPD. We examined the responses of leaf area index (LAI) and evapotranspiration (ET) to VPD along spatial gradients of soil and atmospheric water availability, and further explored the roles of plant water-use strategies and atmospheric moisture conditions using mediation analyses.

**Results:**

Our results showed that vegetation responses of LAI and ET to VPD varied significantly along gradients of soil water content (SWC) and relative humidity (RH). When SWC and RH increased beyond critical thresholds, vegetation responses to VPD shifted from negative to positive for both LAI and ET. These transition patterns were closely associated with spatial variations in plant water-use strategies. Mediation analyses further revealed that changes in ET responses to VPD, jointly regulated by SWC and plant water-use strategies, were associated with variations in atmospheric moisture conditions, which in turn influenced vegetation responses to VPD. Specifically, in water-limited regions, conservative plant water-use strategies and low soil moisture were associated with reduced ET responses under increasing VPD, potentially contributing to lower atmospheric humidity and constrained vegetation productivity. In contrast, in water-rich regions, higher soil moisture and more water-intensive strategies were associated with positive ET responses to VPD, which may help maintain relatively humid atmospheric conditions and less constrained vegetation productivity.

**Discussion:**

Overall, we propose an integrated observational framework to reconcile spatially divergent vegetation responses to VPD, highlighting the importance of soil water availability, atmospheric moisture conditions, and plant water-use strategies in regulating vegetation–climate interactions. These findings may improve our understanding of ecosystem responses to ongoing climate change.

## 1 Introduction

The global atmospheric vapor pressure deficit (VPD) has increased since the late 1990s and is projected to continue rising throughout the 21st century (Fang et al., 2022; Yuan et al., 2019). VPD plays a fundamental role in regulating plant–water relations and is increasingly recognized as an important driver of vegetation dynamics at regional and global scales (Grossiord et al., 2020). Prolonged exposure to high VPD has been associated with large-scale tree mortality (Williams et al., 2013) and wildfire occurrence (Seager et al., 2015), highlighting its ecological significance. Accordingly, negative vegetation responses to elevated VPD have been widely reported in observational studies, particularly in water-limited ecosystems (Fu et al., 2022a; Liu et al., 2020; Chen et al., 2021a; Ding et al., 2018). In contrast, recent studies have also reported positive vegetation responses to increasing VPD in water-abundant regions, such as subtropical and tropical forests (Chen et al., 2021a). These contrasting patterns suggest that vegetation responses to VPD are strongly modulated by environmental water availability. However, the mechanisms underlying these spatially divergent responses remain insufficiently understood at the global scale. Given the important role of vegetation in regulating land–atmosphere interactions, this knowledge gap introduces uncertainty into projections of future climate–carbon cycle feedbacks.

Divergent vegetation responses to VPD are closely linked to how evapotranspiration (ET) responds to atmospheric water demand across spatial gradients. As VPD increases, changes in ET may feed back to the atmosphere and subsequently influence atmospheric moisture conditions (Martin et al., 2010; Seneviratne et al., 2010; Vicente-Serrano et al., 2018). On the one hand, a reduced ET response to increasing VPD has been reported in many ecosystems (Ball et al., 1987), which may contribute to atmospheric drying. For example, decreases in atmospheric relative humidity (RH) have been observed in regions such as Australia, partly attributed to reductions in ET (Simmons et al., 2010). Under such conditions, vegetation photosynthesis may decline because stomatal conductance is often constrained by dry atmospheric conditions (Chen et al., 2022; Niu et al., 2008; Rogiers et al., 2012). On the other hand, in some regions, increasing atmospheric water demand may enhance ET under high VPD conditions (Penman, 1948). Such a positive response may help maintain relatively moist atmospheric conditions, thereby sustaining vegetation productivity, albeit at the cost of increased water loss (Massmann et al., 2019). Therefore, explicitly characterizing the direction and magnitude of ET responses to VPD may improve our understanding of spatially divergent vegetation responses.

The response of ET to increasing VPD is likely regulated by soil moisture availability and plant water-use strategies across space. Under increasing VPD, soil water availability may directly regulate soil evaporation (Es) in response to enhanced atmospheric demand (Seneviratne et al., 2010). When soil moisture is sufficient, Es may increase with VPD; in contrast, under dry soil conditions, Es is often constrained (Martin et al., 2010; Seneviratne et al., 2010). In addition, plants may adopt different water regulation strategies under increasing atmospheric demand, ranging from conservative water use with restricted transpiration rates (Et) to more acquisitive strategies that allow higher Et (Konings and Gentine, 2017; Massmann et al., 2019). In general, a water-intensive strategy is associated with a positive ET response to VPD in water-rich regions (Helbig et al., 2020; Massmann et al., 2019), whereas water-conservative strategies, typically observed in dry shrubs and grasslands, are often associated with reduced ET under high VPD (Chen et al., 2021a; Massmann et al., 2019). Overall, global observations suggest that ET tends to increase with atmospheric water demand in water-rich regions, while decreasing responses are more common in water-limited regions (Zhao et al., 2022).

Vegetation responses to VPD exhibit substantial spatial heterogeneity across the globe (He et al., 2022; Liu et al., 2020; Yuan et al., 2019). However, the mechanisms underlying these divergent responses remain insufficiently explored. In this study, we addressed this gap using global satellite-derived leaf area index (LAI) and ET datasets. We first quantified spatial patterns of ET and LAI responses to VPD, as well as plant water-use strategies along soil and atmospheric moisture gradients. We then examined whether spatial variability in vegetation responses to VPD could be explained by plant water-use strategies. Finally, we proposed a mechanistic framework linking SWC and plant water-use strategy to ET responses to VPD, atmospheric humidity (RH), and LAI responses, thereby providing a conceptual basis for reconciling divergent vegetation responses across global water gradients.

## 2 Materials and methods

To isolate the biophysical responses of vegetation to VPD, we focused on natural ecosystems in this study. Cropland areas were excluded because their vegetation productivity and water–carbon exchanges are strongly regulated by human management practices (e.g., irrigation and fertilization), which can substantially modify soil moisture conditions and plant water use, thereby confounding the direct climatic controls of VPD. Accordingly, all analyses should be interpreted as representing natural vegetation systems rather than the entire terrestrial biosphere.

### 2.1 Satellite data

We used the Global Land Surface Satellite (GLASS) LAI product to characterize vegetation dynamics. The GLASS LAI dataset was derived from Advanced Very High Resolution Radiometer (AVHRR) observations for the period 1981–1999 and Moderate Resolution Imaging Spectroradiometer (MODIS) observations for 2000–2015 (Xiao et al., 2016). The dataset was consistent with MODIS LAI and GEOV1 products in terms of spatial patterns (Xiao et al., 2016). The original spatial and temporal resolutions of GLASS LAI were 0.05° and 8-day, respectively.

ET data were obtained from the GLASS ET product (8-day, 0.05°), which was generated using a multi-model ensemble framework based on Bayesian model averaging. This product integrated five process-based ET algorithms, including MODIS ET, revised remote-sensing-based Penman–Monteith ET, Priestley–Taylor-based ET, modified satellite-based Priestley–Taylor ET, and the semi-empirical Penman ET model from the University of Maryland (Yao et al., 2014).

Monthly climate variables, including air temperature (Ta), precipitation, potential evapotranspiration (PET), and actual vapor pressure (AVP), were obtained from the CRU TS v4.04 dataset (0.5° × 0.5°) (Harris et al., 2020). Based on these variables, we calculated key atmospheric indicators in this study. Saturation vapor pressure (SVP) was first derived using standard formulations (Equations 1, 2), and VPD was subsequently calculated following Yuan et al. (2019).

(1) $$SVP=0.611\times \exp\left(\frac{17.27\times Ta}{237.3+Ta}\right)$$

(2) VPD=SVP−AVP

The aridity index (AI) was defined as the ratio of precipitation to PET, and used to classify regions into arid and semi-arid (AI < 0.5), subhumid (0.5 ≤ AI < 0.65), and humid (AI ≥ 0.65) climates (Lian et al., 2021). RH was calculated as the ratio of AVP to SVP. Monthly solar radiation data were obtained from the ERA5 reanalysis dataset (0.1° × 0.1°) (Hersbach et al., 2020), and root-zone SWC was derived from the GLEAM v3.5a product (0.25° resolution) (Martens et al., 2017). All datasets were resampled to a unified spatial resolution of 0.5° using the nearest-neighbor method and aggregated to a monthly temporal scale for the period 1982–2015.

### 2.2 Spatial water gradient

RH was used to characterize atmospheric moisture conditions across space. Previous studies have suggested that vegetation responses to VPD may not significantly decline when changes in AVP closely track saturation vapor pressure (SVP), resulting in relatively stable RH conditions (Cheng et al., 2022; Yuan et al., 2019). Under such a supply–demand balance of atmospheric moisture, the air may remain relatively moist even under high VPD conditions. In contrast, more pronounced negative vegetation responses to VPD have been observed when increases in SVP are not accompanied by corresponding increases in AVP, leading to reduced RH (Cheng et al., 2022; Yuan et al., 2019). In such cases, the atmospheric water supply–demand balance may be disrupted under increasing VPD, potentially resulting in drier atmospheric conditions. Therefore, variations in RH may provide a useful indicator for identifying spatial transition patterns in vegetation responses to VPD. In addition, SWC was used to represent soil moisture conditions. To examine potential transition thresholds in vegetation responses to VPD, SWC and RH were both classified into six categories: 0–0.22, 0.22–0.27, 0.27–0.32, 0.32–0.37, 0.37–0.42, and ≥ 0.42 m³ m^-^³ for SWC; and 0–40%, 40–50%, 50–60%, 60–70%, 70–80%, and ≥ 80% for RH.

### 2.3 Plant water-use strategy

A theoretical framework for estimating ET responses to increasing VPD was developed based on the Penman–Monteith equation, combined with semi-empirical optimal stomatal regulation theory and intrinsic water-use efficiency (uWUE) (Massmann et al., 2019). The partial derivative ∂ET/∂VPD (Equation 3) was used as an indicator of plant water-use strategy across space. Higher values of ∂ET/∂VPD (i.e., ∂ET/∂VPD > 0) indicate that ET increases in response to rising VPD, suggesting a more water-intensive strategy characterized by relatively open stomata. In contrast, lower values of ∂ET/∂VPD (i.e., ∂ET/∂VPD < 0) indicate reduced ET under increasing VPD, reflecting a more water-conservative strategy associated with stomatal regulation and reduced water loss (Massmann et al., 2019).

(3) $$\frac{\partial ET}{\partial VPD}=\frac{c_{p}}{R_{air}}-\frac{\gamma \times c_{a}}{1.6\times R\times uWUE}\times \left(\frac{2\times g_{1}+\sqrt{VPD}}{2\times \left(g_{1}+\sqrt{VPD}\right)}\right)$$

Where *c_p_* is the specific heat capacity of air at constant pressure (1004.0 J·K^-1^·kg^-1^), *R_air_* is the gas constant of air (288.0 J·K^-1^·kg^-1^), *γ* is the psychometric constant (64.5 Pa/K), *c_a_* is CO_2_ concentration (400.0 μmol CO_2_/mol air), and *R* is the universal gas constant (8.314 J·mol^-1^·K^-1^) (Massmann et al., 2019). uWUE was calculated by global GLASS ET, GLASS gross primary productivity (GPP), and VPD (Zhou et al., 2014). *g_1_* values in multiple plant types were estimated by leaf gas exchange and eddy covariance observations originating from a previous work (Medlyn et al., 2017) (**Supplementary Table 1**). After assessing *g_1_* based on the MODIS land vegetation cover classification product (sixth edition), we matched *g_1_* with vegetation cover classifications and mapped a global distribution of ∂ET/∂VPD.

In addition, the response of Et to VPD was used as an ecosystem-scale proxy to characterize water-use strategies across space. It should be noted that this metric integrates both plant transpiration and non-stomatal evaporation components (e.g., soil evaporation and moss evaporation), and therefore reflects ecosystem-level water-use behavior rather than purely plant physiological regulation. Positive Et responses to VPD were interpreted as a water-intensive strategy, indicating that plants tended to maintain stomatal openness to sustain carbon uptake at the expense of higher water loss. In contrast, negative Et responses to VPD were interpreted as a water-conservative strategy, reflecting partial stomatal closure under increasing atmospheric demand. Et was simulated using the Penman–Monteith–Leuning (PML-V2) model (Zhang et al., 2019), which couples a photosynthesis module and a canopy stomatal conductance scheme with the Penman–Monteith energy balance framework.

### 2.4 Statistical analyses

**Figure 1:**
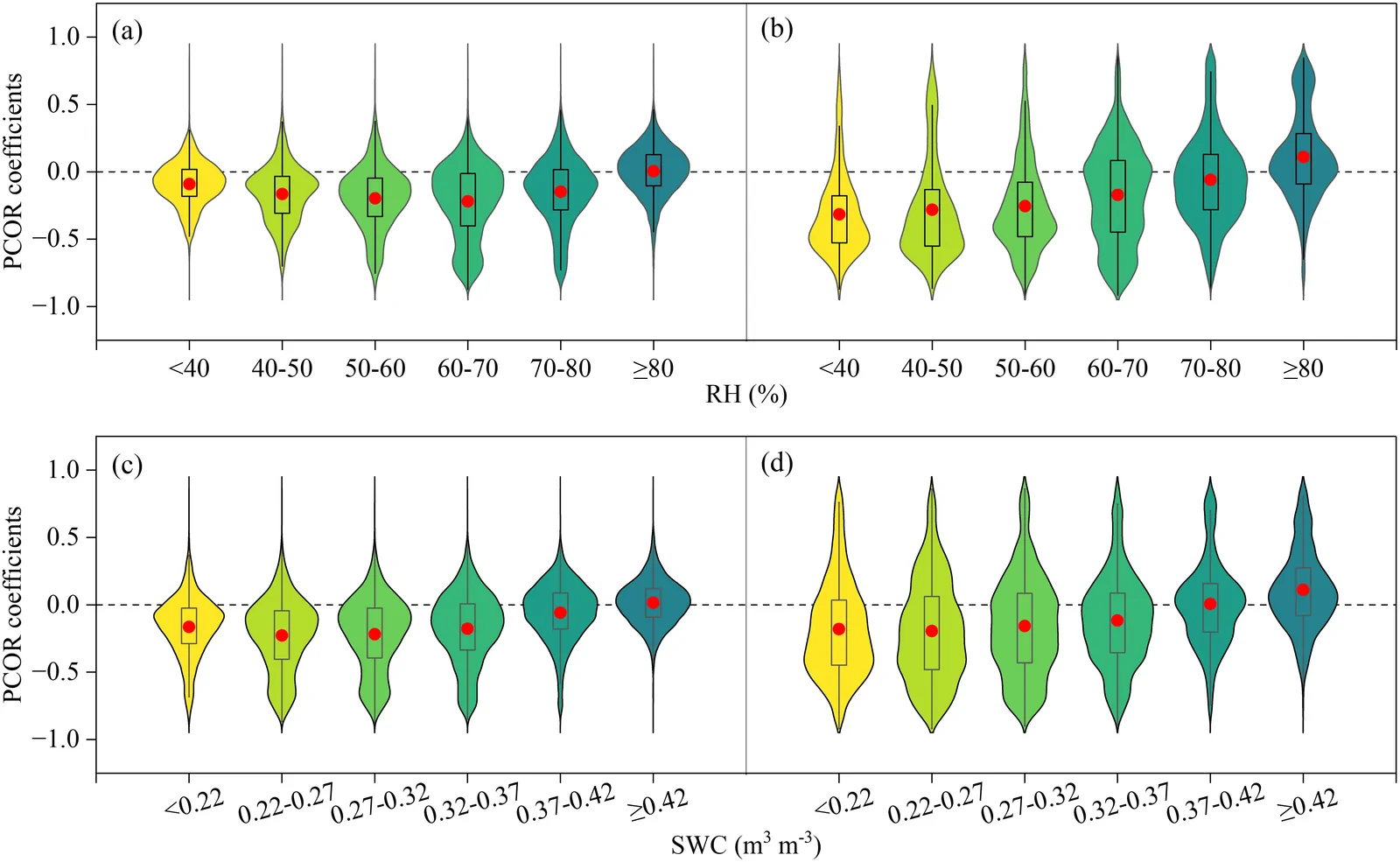
Statistical distribution of the coefficients of PCOR_LAI vs. VPD_ **(a, c)** and PCOR_ET vs. VPD_ **(b, d)** along the spital water gradients of RH **(a, b)** and SWC **(c, d)**. Red dots indicate the mean values of the coefficients of PCOR_LAI vs. VPD_ and PCOR_ET vs. VPD_ **(a−d)**.

Partial correlation analysis was conducted to quantify the relationships between VPD and LAI, ET, and Et, denoted as PCOR_LAI–VPD_, PCOR_ET–VPD_, and PCOR_Et–VPD_, respectively. Analyses were performed for the growing seasons during 1982–2015, while controlling for Ta, solar radiation, and precipitation. Partial correlation coefficients were calculated using the pcor.test function in the ppcor package in R 4.2.2. The growing season was defined as months in which GPP exceeded 20% of its annual maximum for each grid cell. Validation using six ChinaFLUX sites, including grassland, shrubland, and forest ecosystems, indicated that this definition adequately captured seasonal vegetation productivity (**Figure 1**; **Supplementary Table 2**).

Logistic regression and receiver operating characteristic (ROC) analysis were applied to evaluate the relationship between plant water-use strategy (represented by PCOR_Et–VPD_) and vegetation responses to VPD (PCOR_LAI–VPD_ and PCOR_ET–VPD_) across space. For this purpose, grid-cell-based correlation coefficients (PCOR_LAI–VPD_, PCOR_ET–VPD_, and PCOR_Et–VPD_) were extracted as analytical variables. PCOR_LAI–VPD_ and PCOR_ET–VPD_ were treated as dependent variables and converted into binary categories (0: negative response; 1: positive response). PCOR_Et–VPD_ was used as the predictor variable. Logistic regression was performed to test the association between plant water-use strategy and vegetation response types. When significant relationships were detected, ROC curves were used to determine optimal threshold values. The optimal cut-off values of PCOR_Et–VPD_ were defined as spatial transition thresholds separating positive and negative vegetation responses to VPD. Model performance was evaluated using the area under the ROC curve (AUC, 0–1). All analyses were conducted using the roc function in the pROC package in R 4.2.2.

Mediation analysis was applied to evaluate potential causal pathways linking SWC and plant water-use strategy to LAI responses to VPD through ET responses and RH. The conceptual framework assumed a sequential pathway: SWC and plant water-use strategy → ET response to VPD → RH → LAI response to VPD. For each grid cell, ∂ET/∂VPD, SWC, RH, and correlation coefficients (PCOR_LAI–VPD_, PCOR_ET–VPD_, PCOR_Et–VPD_) were extracted as input variables. In the mediation model, SWC, PCOR_Et–VPD_, and ∂ET/∂VPD were treated as independent variables, PCOR_LAI–VPD_ was treated as the dependent variable, and RH and PCOR_ET–VPD_ were treated as mediators. Mediation analyses were performed using functions from the bruceR package in R 4.2.2.

## 3 Results

### 3.1 Divergent responses of LAI and ET to VPD along spatial water gradients

**Figure 2:**
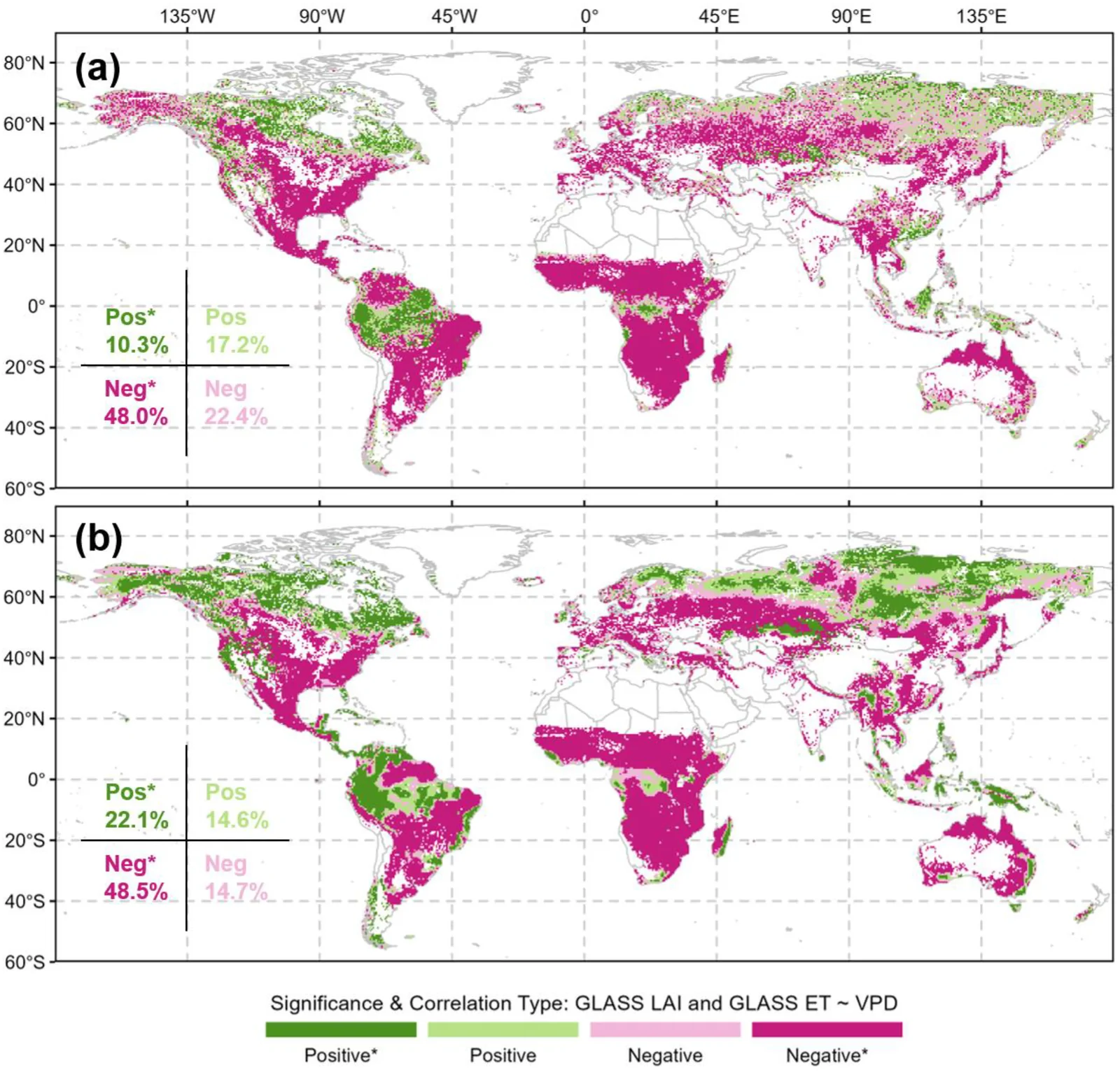
Spatial distributions of relationships between GLASS LAI **(a)**, GLASS ET **(b)**, and VPD **(c)**. Relative frequency (%) distributions of significant negative correlations (Neg*), significant positive correlations (Pos*), insignificant negative correlations (Neg), and insignificant positive correlations (Pos). Gray areas were excluded from the analysis, mainly including croplands and barren land (vegetation cover < 10%).

Vegetation responses to VPD exhibited pronounced spatial heterogeneity and were broadly consistent with global water availability gradients. Partial correlation analyses showed that LAI and ET were significantly and negatively correlated with VPD in 48.0% and 48.5% of vegetated areas, respectively (**Figure 2**; p < 0.05). These negative responses were predominantly distributed in arid and semi-arid regions, which accounted for more than 40% of the significant negative correlations (**Figure 2**). Negative responses were also observed in seasonally dry ecosystems, including Mediterranean regions and tropical savannas, even in some areas classified as humid (**Figure 2**). In contrast, positive correlations between VPD and LAI and ET were observed in 27.5% and 36.7% of vegetated areas, respectively (significant proportions: 10.5% and 22.1%; p < 0.05) (**Figure 2**). These positive responses were mainly distributed in boreal water-rich regions (including tundra and northern peatlands), as well as boreal, subtropical, and tropical forest ecosystems (**Figure 2**).

RH and SWC further quantify the divergent responses of LAI and ET to VPD across spatial water gradients. For example, when global RH was divided into six bins, the mean coefficients of PCOR_LAI–VPD_ and PCOR_ET–VPD_ shifted from negative values (PCOR_LAI–VPD_: −0.18; PCOR_ET–VPD_: −0.15) to positive values (PCOR_LAI–VPD_: 0.004; PCOR_ET–VPD_: 0.11) at an RH threshold of 80% (**Figures 1a, b**). Similarly, at an SWC threshold of 0.42 m^3^ m^−3^, the mean coefficients of PCOR_LAI–VPD_ and PCOR_ET–VPD_ increased from −0.17 and −0.13 to 0.02 and 0.11, respectively (**Figures 1c, d**). These results highlight that spatial water gradients can effectively reveal the divergent responses of LAI and ET to VPD.

### 3.2 Divergent plant water-use strategies along spatial water gradients

**Figure 3:**
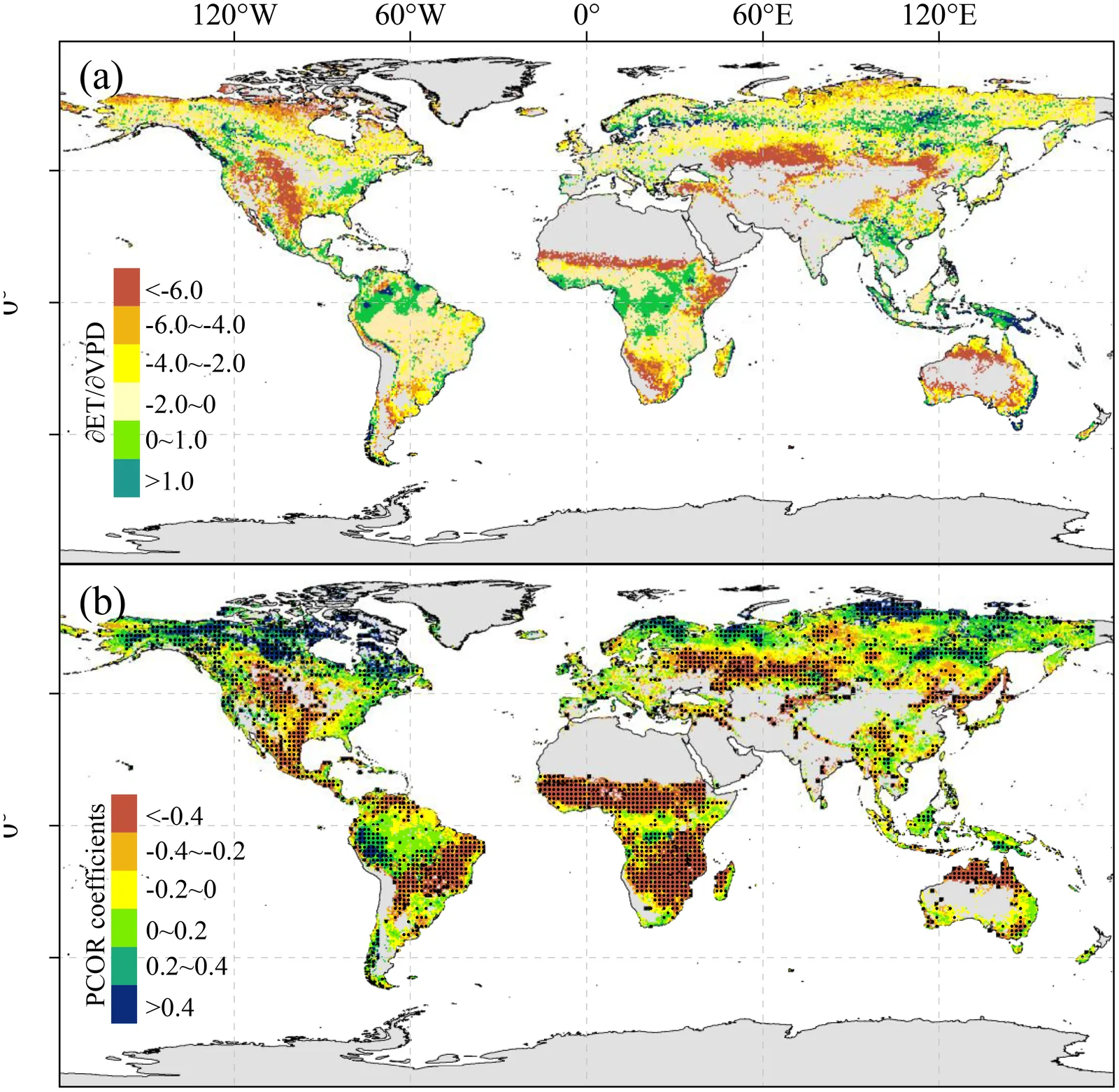
Spatial distribution of ∂ET/∂VPD **(a)** and the coefficient of PCOR_Et vs. VPD_ **(b)**. Black dots indicate significant correlations with *p* < 0.05 **(b)**. Gray areas were removed in our study, mainly including the cropland and barren land (vegetation cover < 10%).

**Figure 4:**
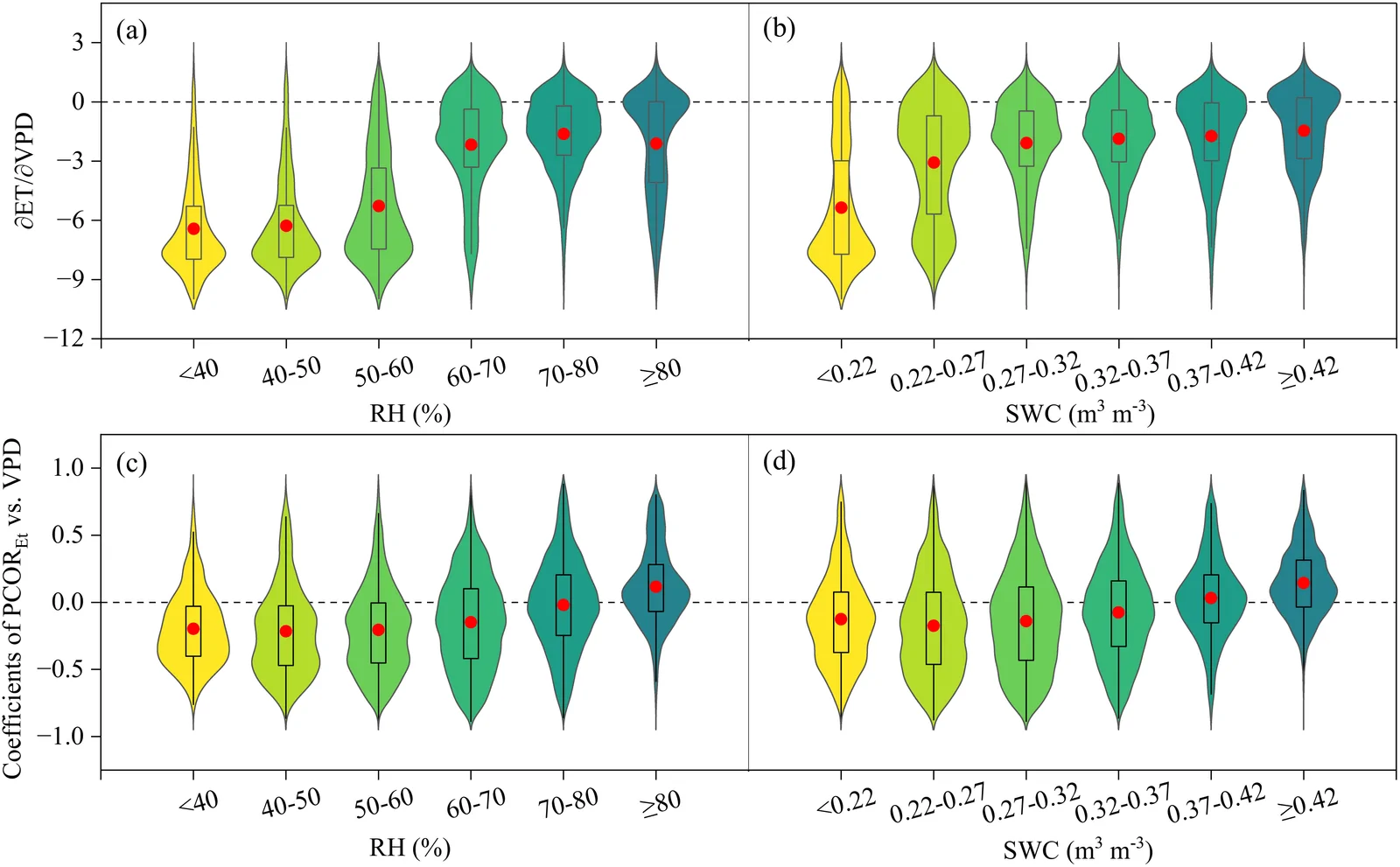
Statistical distributions of ∂ET/∂VPD **(a, b)** and the coefficient of PCOR_Et vs. VPD_ **(c, d)** along the spatial water gradients of RH **(a, c)** and SWC **(b, d)**. Red dots indicate the mean values of ∂ET/∂VPD and the coefficients of PCOR_Et vs. VPD_ **(a−d)**.

Spatial water gradients also exhibited clear patterns in plant water-use strategies. Higher values of ∂ET/∂VPD (∂ET/∂VPD > 0), together with significant positive PCOR_Et–VPD_ values (p < 0.05), were mainly distributed in water-rich boreal regions (tundra and northern peatlands), as well as boreal, subtropical, and tropical forest ecosystems (**Figures 3a, b**). In contrast, lower values of ∂ET/∂VPD (∂ET/∂VPD < 0) and significant negative PCOR_Et–VPD_ values (p < 0.05) were primarily observed in arid and semi-arid regions, including the Eurasian steppes and North American prairies, as well as seasonally dry Mediterranean and tropical savanna ecosystems (**Figures 3a, b**). Furthermore, when SWC and RH were divided into six classes, ∂ET/∂VPD showed increasing trends along both soil and atmospheric moisture gradients (**Figures 4a, b**). At the identified transition thresholds (SWC ≈ 0.42 m^3^ m^−3^ and RH ≈ 80%), mean PCOREt–VPD values shifted from negative to positive (SWC: −0.09 to 0.14 m^3^ m^−3^; RH: −0.11 to 0.12) (**Figures 4c, d**).

### 3.3 Plant water-use strategy and SWC as major contributors to divergent LAI responses to VPD

**Figure 5:**
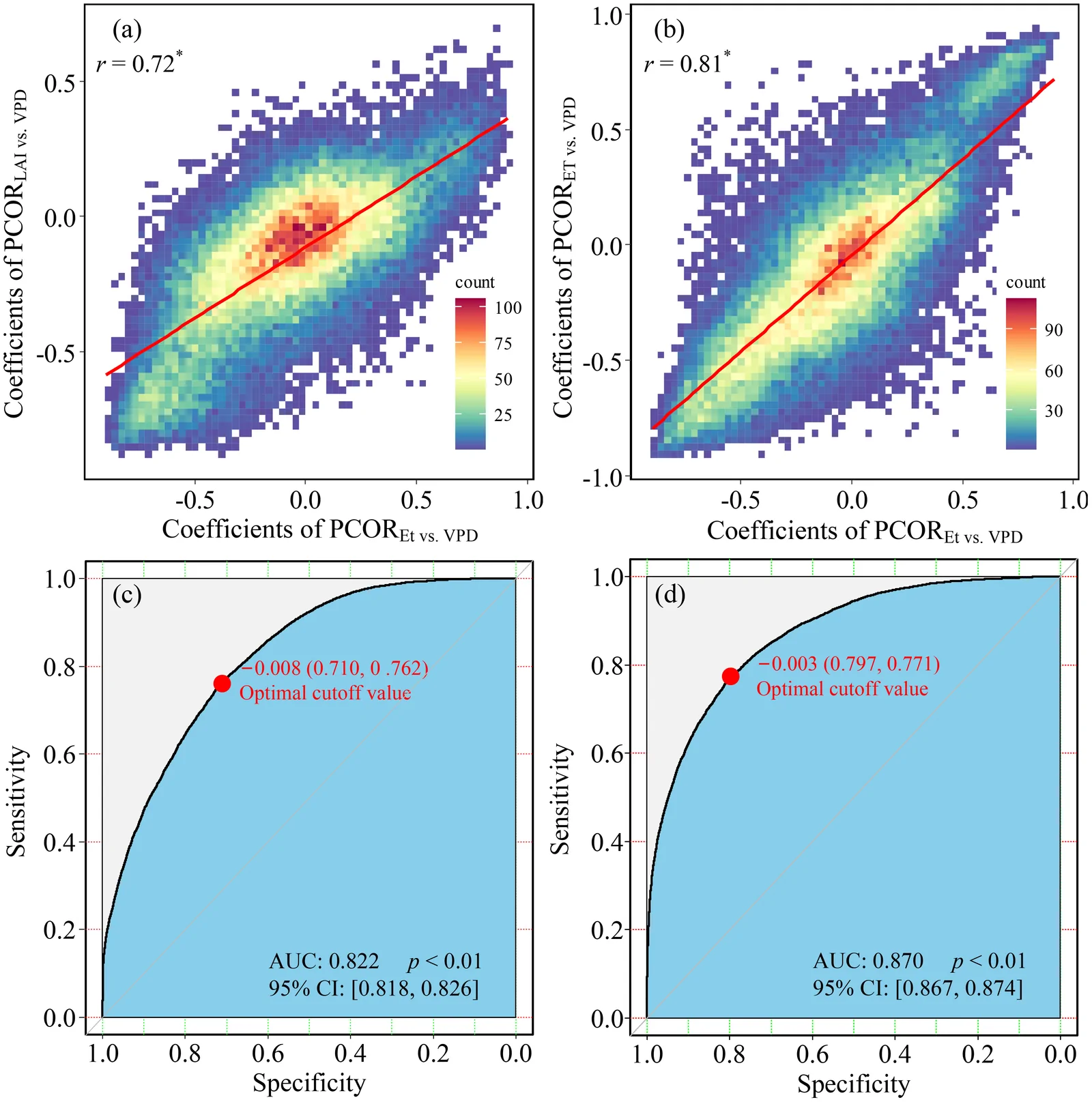
Correlations **(a, b)** and ROC curve analyses **(c, d)** of the coefficient of PCOR_Et vs. VPD_ with the coefficients of PCOR_LAI vs. VPD_ **(a, c)** and PCOR_ET vs. VPD_ **(b, d)**. In panels **(a, b)**, *r* denotes Pearson correlation coefficients, and all correlations are significant at the 0.05 level. Panels **(c, d)** present receiver operating characteristic (ROC) curves evaluating the ability of the coefficient of PCOR_Et vs. VPD_ to distinguish spatial transitions from negative to positive responses in PCOR_LAI vs. VPD_ and PCOR_ET vs. VPD_, respectively. The blue shaded area represents the area under the curve (AUC). The AUC values and their 95% confidence intervals (CI) are reported in each panel. Red dots indicate the optimal cutoff values, with corresponding sensitivity and specificity shown in parentheses. Based on the ROC results, the spatial transitions from negative to positive responses in PCOR_LAI vs. VPD_ and PCOR_ET vs. VPD_ can be well captured by threshold values of −0.008 and −0.003, respectively.

Spatial patterns of plant water-use strategy were closely associated with the divergent responses of ET and LAI to VPD. The coefficient of PCOR_Et–VPD_ was positively correlated with both PCOR_ET–VPD_ and PCOR_LAI–VPD_, with correlation coefficients exceeding 0.70 (**Figures 5a, b**; p < 0.05). Logistic regression analyses further supported these relationships (**Supplementary Table 3**), indicating that plant water-use strategy was spatially consistent with vegetation responses to VPD. ROC analyses showed strong predictive performance (AUC > 0.8), with more than 70% of positive and negative responses of LAI and ET correctly classified using PCOR_Et–VPD_ (**Figures 5c, d**). Based on ROC-derived thresholds, transitions in PCOR_LAI–VPD_ and PCOR_ET–VPD_ from negative to positive were well captured by PCOR_Et–VPD_ thresholds of approximately −0.008 and −0.003, respectively (**Figures 5c, d**). These results indicated that high and low values of the ecosystem-scale water-use metric were spatially consistent with positive and negative vegetation responses to VPD, respectively. Plant water-use strategy and SWC were further associated with LAI responses to VPD through their relationships with ET responses and RH. Mediation analyses indicated that indirect pathways involving SWC, ∂ET/∂VPD, and PCOR_Et–VPD_ were statistically significant across multiple SWC gradients (**Table 1**; p < 0.05).

**Table 1:** Summary of the mediation effect analyses under multiple SWC gradients.

Groups	Independent variables	Indirect effects	*P* values
SWC < 0.42 m^3^m^-3^	SWC	0.076	<0.001
	PCOR_Et vs. VPD_	0.308	<0.001
	∂ET/∂VPD	0.107	<0.001
SWC ≥ 0.42 m^3^m^-3^	SWC	0.075	<0.001
	PCOR_Et vs. VPD_	0.233	<0.001
	∂ET/∂VPD	0.037	0.010
Total SWC	SWC	0.124	<0.001
	PCOR_Et vs. VPD_	0.307	<0.001
	∂ET/∂VPD	0.130	<0.001

## 4 Discussion

### 4.1 Negative responses of LAI and ET to VPD

Negative responses of LAI and ET to VPD were predominantly observed in temperate grasslands, where water limitation is a key constraint. These patterns were consistent with previous experimental results from temperate grassland ecosystems in northern China (Niu et al., 2008). In these regions, limited precipitation (<500 mm yr^-^¹), strong solar radiation, and high wind speed contribute to low soil water availability and weak water retention capacity (Liu et al., 2023). Although Es can contribute substantially to ET, its response to increasing VPD may still be constrained under dry soil conditions, as indicated by negative Es responses (**Supplementary Figure 2**). Additionally, vegetation in these ecosystems is typically characterized by shallow root systems, which restrict access to deeper soil moisture under dry conditions. Consequently, water-conservative strategies tend to dominate, as reflected by lower ∂ET/∂VPD and negative PCOR_Et–VPD_ values. During periods of high atmospheric demand, stomatal closure may occur to reduce hydraulic risk, thereby limiting carbon uptake and vegetation productivity (Meinzer et al., 2016). Similar patterns were also observed in seasonally dry regions such as Mediterranean ecosystems and tropical savannas. These ecosystems often exhibit increased isohydric behavior during dry seasons, reflecting heightened sensitivity to hydraulic stress (Konings and Gentine, 2017; Jin et al., 2023).

### 4.2 Positive responses of LAI and ET to VPD

Positive responses of LAI and ET to VPD were mainly observed in boreal forests, subtropical forests, and tropical forests. In these ecosystems, energy limitation rather than water limitation is often the dominant constraint on vegetation processes. Although mean annual precipitation in some boreal regions is relatively low, high soil water-holding capacity due to organic-rich soils and low evaporative losses help maintain sufficient water availability (Stack et al., 2020). Under these conditions, plant water uptake may be supported by deeper or more stable soil water sources, allowing Et to increase with VPD (Chen et al., 2021a; Seneviratne et al., 2010). In subtropical and tropical forests, light limitation often dominates ecosystem functioning. Therefore, maintaining stomatal openness under increasing VPD may be favorable for carbon gain despite increased water loss (Konings and Gentine, 2017). These characteristics are consistent with higher ∂ET/∂VPD and positive PCOR_Et–VPD_ values observed in these regions. In addition, evaporative cooling associated with Et may help regulate leaf temperature and mitigate heat stress, potentially supporting photosynthetic activity under high VPD conditions (Choat et al., 2012; Crawford et al., 2012). In boreal peatlands and tundra, Es may play a particularly important role in total ET. Shallow water tables, snowmelt inputs, and permafrost thaw can enhance water availability at the surface, contributing to increased evaporation under higher VPD (Helbig et al., 2016; Qi et al., 2020). Moss-dominated ecosystems may further enhance ET responses due to their high water storage capacity and limited physiological control over water loss (Williams and Flanagan, 1996; Heijmans et al., 2004). These processes may contribute to relatively stable or slightly increasing atmospheric humidity conditions, consistent with observed RH trends (Vicente-Serrano et al., 2018).

### 4.3 Limitations and future directions

Several limitations should be considered. First, estimation of plant water-use strategy may be uncertain in ecosystems where parameterization of g_1_ is limited, particularly in peatlands and tundra ecosystems (Medlyn et al., 2017; Lin et al., 2018). Second, the use of ∂ET/∂VPD as a proxy for plant water-use strategy assumes that ET is primarily controlled by Et. This assumption may not hold in ecosystems with substantial Es contributions, such as wetlands and cold regions. Although the spatial pattern of ∂ET/∂VPD generally aligns with PCOR_Et–VPD_ in most regions, deviations in high-latitude ecosystems suggest potential uncertainty in physiological interpretation. Third, the framework primarily focused on water-related controls, while other factors such as temperature, nutrient availability, elevated CO_2_, and vegetation composition may also influence vegetation responses to VPD. In addition, variables such as RH are jointly affected by large-scale atmospheric circulation, moisture advection, and oceanic moisture transport, and therefore should not be interpreted solely as a local consequence of land evapotranspiration feedbacks. Finally, temporal dynamics were not explicitly considered in this framework. However, vegetation responses to VPD may vary over time under changing climate conditions, potentially affecting ecosystem water balance and increasing risks of flash droughts (Zhao et al., 2022). In addition, it should be noted that the identified RH (~80%) and SWC (~0.40–0.42 m^3^ m^-3^) transition thresholds are empirical values derived from the global gridded datasets and binning approach used in this study. These thresholds may therefore vary with dataset choice, spatial resolution, biome composition, and methodological settings, and should not be interpreted as universal ecological tipping points. Future studies incorporating multi-temporal and process-based modeling approaches are needed to better resolve these dynamics.

## 5 Conclusions

Increasing atmospheric VPD was associated with divergent vegetation responses across global water gradients, ranging from negative to positive effects on vegetation productivity and evapotranspiration. Our results suggest that these contrasting responses are linked to differences in soil moisture conditions and plant water-use strategies, which may regulate evapotranspiration responses and atmospheric humidity conditions. In water-limited regions, reduced soil moisture and conservative plant strategies were associated with reduced ET responses under high VPD, potentially contributing to lower atmospheric humidity and constrained vegetation productivity. In contrast, in water-rich regions, higher soil moisture and more water-intensive strategies were associated with enhanced ET responses under increasing VPD, potentially helping maintain atmospheric humidity and supporting vegetation productivity. The transition between negative and positive responses was associated with empirical thresholds of approximately 0.42 m^3^ m^–3^ SWC and 80% RH. These thresholds should be interpreted as dataset-dependent transition points. Overall, this study provides a unified observational framework for understanding spatially divergent vegetation responses to VPD and highlights the importance of background water availability in regulating land–atmosphere interactions under increasing atmospheric water demand.

## Data Availability

Publicly available datasets were analyzed in this study. This data can be found here: GLASS ET/LAI/GPP were obtained from http://www.glass.umd.edu/. GLEAM v3.5a SWC was available at https://www.gleam.eu/. CRUST 4.04 precipitation/Ta/AVP/were obtained from http://climexp.knmi.nl/selectfield_obs2.cgi?id=someone/somewhere. ERA5 SR was obtained from https://cds.climate.copernicus.eu/#!/home. PML v2 soil evaporation and transpiration were provided by National Tibetan Plateau Data Center (http://data.tpdc.ac.cn). Datasets of six ChinaFLUX sites were obtained from http://www.chinaflux.org/.
