# Supplementary material for: Vegetation productivity responses to increased atmospheric vapor pressure deficit across the globe
Source: Front Plant Sci. 2026 Aug 12;17:1886959. doi: 10.3389/fpls.2026.1886959 (PMC13506881; doi:10.3389/fpls.2026.1886959)
Supplement: Supplementary file 1 [file DataSheet1.docx]

**Supplementary Materials for**

**Vegetation productivity responses to increased atmospheric vapor pressure deficit across the globe**

Meiou Qin^1^, Xiaotao Wang^1^, Mengyuan Zhu^2^, Peng Jiang^3^*, Rihong Wen^4^ *, Binshuo Liu^5^, Xingyang Li^6^

^1^ Liaoning Climate Center, Shenyang 110016, China

^2^ Liaoning Meteorological Service Center, Shenyang 110016

^3^ Liaoning Agricultural Meteorology and Satellite Remote Sensing Center, Shenyang 110016, China

^4^ Institute of Atmospheric Environment, China Meteorological Administration, Shenyang 110016, China

^5^ Liaoning Ecological Meteorology and Satellite Remote Sensing Center, Shenyang 110016, China

^6^ Tianjin Jinghai District Meteorological Bureau, Tianjin 301600

^*^Corresponding authors: Peng Jiang (jiangpenglnqx@iaesy.cn); Rihong Wen (wenrihong@iaesy.cn)

Meiou Qin and Xiaotao Wang contributed equally to this work and share first authorship

 **Figure S1** Dynamics of GPP (mean±1 SD) during the growing seasons in six ChinaFLUX sites. Shades of grey indicated the month that GPP was higher than 20 % of the monthly peak values during a year, that is, growing seasons.

**Figure S2** Spatial distribution of the coefficient of PCOR_Es vs. VPD_. Black dots indicate significant correlations with *p* < 0.05. Gray areas were removed in our study, mainly including the cropland and barren land (vegetation cover < 10%).

**Table S1** The descriptions of g1 in this study

| **Types** | **Values** | **Notes** |
| --- | --- | --- |
| ENF | 3.202 | Direct from Medlyn et al., 2017 |
| DNF | 3.202 | Same as ENF |
| EBF | 3.590 | Direct from Medlyn et al., 2017 |
| DBF | 3.681 | Direct from Medlyn et al., 2017 |
| MF | 3.419 | Mean of ENF, EBF and DBF |
| CSH | 4.650 | SHB from Medlyn et al., 2017 |
| OSH | 4.650 | SHB from Medlyn et al., 2017 |
| SAV | 4.225 | SAV from Medlyn et al., 2017 |
| WAV | 4.225 | SAV from Medlyn et al., 2017 |
| GRA | 2.985 | Mean of C3 GRA and C4 GRA from Medlyn et al., 2017 |
| GRO | 3.167 | Mean of C3 GRO and C4 GRO from Medlyn et al., 2017 |
| Others | 3.727 | Mean of above all types |

Evergreen broadleaf forest (EBF), deciduous needleleaf forests (DNF), deciduous broadleaf forest (DBF), evergreen needleleaf forest (ENF), mixed forest (MF), grassland (GRA), croplands (CRO), open shrublands (OSH), closed shrublands (CSH), savannas (SAV), and woody savannas (WSA), others (permanent wetlands, etc.).

**Table S2** The descriptions of the ChinaFLUX sites using in this study

| **Sites** | **Latitude** | [**Longitude**](javascript:;) | **Types** | **Period** | **This study** | **Other studies** |
| --- | --- | --- | --- | --- | --- | --- |
| DX | 30.683 | 91.017 | GRA | 2003-2010 | 5-10 | 5-9 (J. Zhao et al., 2019) |
| DHS | 23.167 | 112.567 | EBF | 2003-2010 | 1-12 | 1-12 (Yu et al., 2008) |
| NMG | 43.633 | 116.700 | GRA | 2004-2008 | 5-9 | 5-10 (Xia, Niu, & Wan, 2009) |
| QYZ | 26.733 | 115.050 | MF | 2003-2010 | 1-12 | 1-12 (Yu et al., 2008) |
| CBS | 42.403， | 28.096 | MF | 2003-2010 | 5-10 | 5-10 (Shi, Hou, Wang, Wang, & Niu, 2018) |
| HB | 37.600 | 101.317 | GRA and OSH | 2003-2010 | 5-9 | 5-10 (L. Zhao et al., 2006) |

Evergreen broadleaf forest (EBF), mixed forest (MF), grassland (GRA), open shrublands (OSH); The column of “This study” and “Other studies” indicated the long growing seasons were observed by our study and other studies, respectively.

**Table S3** Summary the results of logistic regression

|  | Estimate | Std. Error | z value | p value |
| --- | --- | --- | --- | --- |
| PCOR_Et_ vs. PCOR_ET_ | 5.91 | 0.06 | 95.03 | <0.01 |
| PCOR_Et_ vs. PCOR_LAI_ | 4.37 | 0.05 | 85.43 | <0.01 |

PCOR_Et_, PCOR_ET_ and PCOR_LAI_ indicated the coefficients of PCOR_Et vs. VPD_, PCOR_ET vs. VPD_, and PCOR_LAI vs. VPD_, respectively.

**References**

Shi, X., Hou, J., Wang, B., Wang, A., & Niu, S. (2018). Relationship between primary productivity and temperature in broadleaved Pinus koraiensis mixed forest in Changbai Mountains of northeastern China. *Journal of Beijing Forestry University, 40*(11), 49-57. doi:<https://doi.org/10.13332/j.1000-1522.20180275>

Xia, J., Niu, S., & Wan, S. (2009). Response of ecosystem carbon exchange to warming and nitrogen addition during two hydrologically contrasting growing seasons in a temperate steppe. *Global Change Biology, 15*(6), 1544-1556. doi:<https://doi.org/10.1111/j.1365-2486.2008.01807.x>

Yu, G., Zhang, L., Sun, X., Fu, Y., Wen, X., Wang, Q., . . . Liu, Y. (2008). Environmental controls over carbon exchange of three forest ecosystems in eastern China. *Global Change Biology, 14*(11), 2555-2571. doi:<https://doi.org/10.1111/j.1365-2486.2008.01663.x>

Zhao, J., Luo, T., Wei, H., Deng, Z., Li, X., Li, R., & Tang, Y. (2019). Increased precipitation offsets the negative effect of warming on plant biomass and ecosystem respiration in a Tibetan alpine steppe. *Agricultural and Forest Meteorology, 279*, 107761. doi:<https://doi.org/10.1016/j.agrformet.2019.107761>

Zhao, L., Li, Y. N., Xu, S. X., Zhou, H. K., Gu, S., Yu, G. R., & Zhao, X. Q. (2006). Diurnal, seasonal and annual variation in net ecosystem CO2 exchange of an alpine shrubland on Qinghai-Tibetan plateau. *Global Change Biology, 12*(10), 1940-1953. doi:<https://doi.org/10.1111/j.1365-2486.2006.01197.x>
